# Low dose CT-based spatial analysis (CTSA) to measure implant migration after ceramic hip resurfacing arthroplasty (HRA): A phantom study

**DOI:** 10.1177/09544119231153905

**Published:** 2023-02-11

**Authors:** Susannah G Clarke, Kartik Logishetty, Camilla Halewood, Justin P Cobb

**Affiliations:** 1 MSk Lab, Imperial College London, London, UK; 2 Embody Orthopaedic Limited, London

**Keywords:** Biomedical devices, computed tomography (CT) analysis, hip protheses, imaging (medical), implants/prosthetics, photogrammetry/stereo photogrammetry (medical), implant migration

## Abstract

Implant migration is a predictor of arthroplasty survivorship. It is crucial to monitor the migration of novel hip prostheses within premarket clinical investigations. RSA is the gold standard method, but requires calibrated radiographs using specialised equipment. A commercial computed tomography micromotion analysis solution is a promising alternative but is not yet available for use with monobloc ceramic implants. This study aimed to develop and validate a CT-based spatial analysis (CTSA) method for use with ceramic implants. A phantom study was undertaken to assess accuracy and precision. A ceramic hip resurfacing arthroplasty (HRA) and 20 tantalum beads were implanted into a synthetic hip model and mounted onto a 6-degree of freedom motion stage. The hip was repeatedly scanned with a low dose CT protocol, with imposed micromovements. Data were interrogated using a semiautomated technique. The effective radiation dose for each scan was estimated to be 0.25 mSv. For the head implant, precision ranged between 0.11 and 0.28 mm for translations and 0.34°–0.42° for rotations. For the cup implant, precision ranged between 0.08 and 0.11 mm and 0.19° and 0.42°. For the head, accuracy ranged between 0.04 and 0.18 mm for translations and 0.28°–0.46° for rotations. For the cup, accuracy ranged between 0.04 and 0.08 mm and 0.17° and 0.43°. This in vitro study demonstrates that low dose CTSA of a ceramic HRA is similar in accuracy to RSA. CT is ubiquitous, and this method may be an alternative to RSA to measure prosthesis migration.

## Introduction

Aseptic loosening is the most common cause of revision surgery after hip arthroplasty.^1^ Early implant migration can predict long-term revision risk,^2^ and its detection is a crucial metric to assess the safety of novel prostheses prior to their widespread adoption. Roentgen stereophotogrammetric analysis (RSA) can measure three-dimensional (3D) migrations decomposed into translations and rotations, and is the gold standard technique. Because it is highly accurate, RSA can be used as a surrogate measure for implant survival with small sample sizes,^3^ ideal for clinical safety investigations of new implants. Less accurate solutions such as EBRA^4^ offer the benefit of being entirely markerless, and therefore can be utilised retrospectively, but the reduction in accuracy leads to a higher sample size requirement. Despite its exactness, the introduction of the model-based version and recommendations for its use as a screening method for migration,^2,5–8^ RSA has not become widely adopted as part of the premarket safety analysis of new implants. It requires calibrated radiographs taken with specialised equipment, technical expertise, and bespoke analytical software. This is both time-consuming and expensive, and the patient is exposed to a high radiation dose, so RSA has been confined to use in dedicated centres. Computed tomography (CT) is a ubiquitous method to create high-resolution 3D images of voxels of submillimetre size. A commercial solution, computed tomography micromotion analysis (CTMA, Sectra, Linköping, Sweden) is a promising alternative to RSA which uses CT scans to determine movements between implants and bones in a similar fashion to RSA with similar accuracy and precision,^9–12^ but is not yet available for use with all implants.

Traditional RSA involves the attachment and implantation of small radiopaque markers – such as tantalum beads – onto the implant and into the skeleton during surgery, to serve as landmarks. Model-based RSA is considered a more convenient alternative as it does not require beads to be inserted into the implant, although they are still implanted into the skeleton. Model-based RSA has been shown to be adequate to assess clinical migration of acetabular cups,^13^ femoral stems^14^ and resurfacing heads.^15^ In both RSA methods the patient is positioned carefully above a calibration cage before stereographic radiographs are taken to determine the location of the bone markers and to create a three-dimensional frame of reference. The number of markers inserted must be high enough to allow redundancy in case of any unexpected marker movement or bone changes, although this is unlikely to occur significantly within the timeline of a premarket clinical investigation. In traditional RSA, the implant is also located using markers. In model-based RSA the implant is located using reverse-engineered implant models or manufacturer CAD models.^13–15^ The relative position between implants and bones can be calculated. Taking a series of RSA images over time can indicate whether an implant is moving relative to its host bone.

Several groups have demonstrated how CT may be a suitable alternative for assessing migration of implants after hip arthroplasty. Some of these studies have used tantalum beads embedded into implants^10,12^ and others have used a model-based method to measure migration without modifying the implants.^9,16–18^ One of these groups has developed an entirely markerless system^10^ which has the potential to be used in routine clinical practice. In the current study, a semi-automated low dose CT spatial analysis (CTSA) method was developed for assessing migration in a monobloc ceramic hip resurfacing implant within a premarket clinical investigation. Implant material can affect CT acquisition as image artefacts from ceramic implants may distort anatomical features. This system therefore uses bone markers. Studies have shown that the implant design^13^ and CT protocol^16^ affect migration analysis validity. Validation of this method is therefore necessary before its use in a clinical investigation.

The aim of this study was to establish the accuracy, precision and reproducibility of our CTSA method in a monobloc ceramic hip resurfacing through a phantom study.

## Methods

A Phantom study was chosen as the means to assess accuracy, precision and reproducibility of the new CTSA method. Published phantom studies with synthetic bone have been used to assess technique accuracy and precision in femoral stems,^19,20^ acetabular cups^12,21^ and hip resurfacings.^15^ All of these studies used double measurements to assess precision. Some studies used a motion stage to compare calculated accuracy to inputted movements,^12,20^ whilst others used traditional RSA as a gold standard to compare to.^15,19,21^ This study utilised a motion stage.

### Phantom set-up

**Figure 1.**
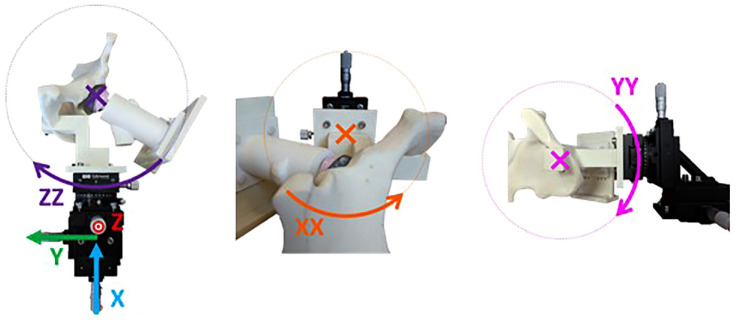
Co-ordinate system for imposed migration on phantom pelvis. The rig is shown set up for measuring cup migration.

A custom phantom with a similar radio-opacity to bone was made from nylon using selective laser sintering to replicate a left hemi-pelvis and proximal femur (Figure 1). Depending on whether the migration of the acetabular cup or femoral head implant was being measured, either the hemi-pelvis or femur was mounted onto a high-precision 6-axis motion stage. This comprised an XYZ 25 mm translation stage with 0.010 mm resolution (model PT3/M), a rotation stage with 0.040° resolution (model PR01/M) (Thorlabs, Inc., Newton, NJ, USA) and a ± 10° goniometer with 0.083° resolution (model 66-536, Edmund Optics, York, UK). It could simulate migration in three directions of translation (superior-inferior, medial-lateral, and anterior-posterior) and around three axes of rotation (flexion/extension, abduction/adduction, and internal/external rotation).

**Figure 2.**
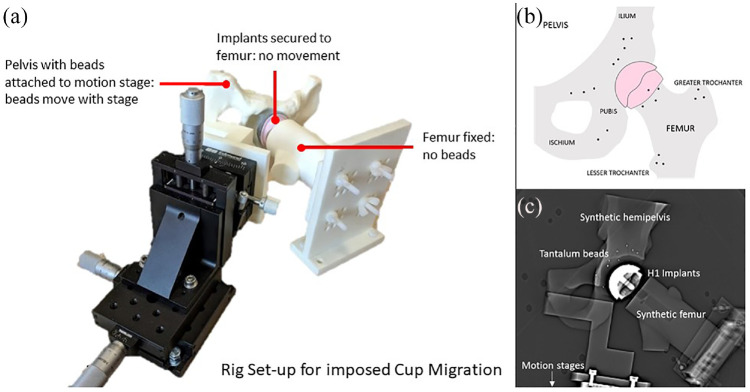
(a) Phantom pelvis mounted on motion stage, (b) Phantom pelvis with tantalum beads inserted into surrounding bone and hip resurfacing arthroplasty head and cup implants and (c) A CT topogram showing appearance and orientation in the CT scanner.

Eighteen 1 mm spherical tantalum beads were inserted and fixed into the periacetabular surface of the hemi-pelvis model and proximal femur model, replicating the distribution for markers in clinical RSA trials (Figure 2(b)). These markers defined two distinct rigid body segments. A cementless 40 mm H1 ceramic femoral head resurfacing implant was glued inside a cementless 47 mm H1 ceramic cup (Embody Orthopaedic Limited, London, UK) to represent the head-cup construct in the clinical setting. To measure cup migration, the head-cup construct was rigidly fixed to the femur model, which was mounted in an anatomical position to the phantom’s base plate (Figure 2(a)). The hemi-pelvis model was mounted to the motion stages. There was sufficient clearance between the cup and the reamed acetabulum of the hemi-pelvis model so that the hemi-pelvis could move freely around the implant construct. Migration was then simulated by moving the hemi-pelvis model and beads around the implants. This was done in lieu of moving the implants relative to the beads as it allowed for the clinically relevant scenario of both implants being present in one scan (Figure 2(c)). Conversely, to measure head migration, the implant construct was rigidly fixed to the hemi-pelvis model, which was mounted in an anatomical position to the phantom’s base. The femur model was mounted to the motion stages with sufficient clearance between the head implant and the femur for small movement; migration was simulated by moving the femur model and beads around the implant construct (Figure 2).

### CT protocol

**Figure 3.**
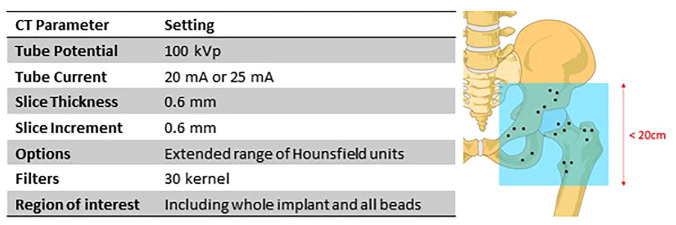
Low dose CT protocol for hip prosthesis migration analysis.

The phantom was orientated in a CT scanner (Somatom Definition AS+; Siemens, Munich, Germany) to mimic a clinical examination. The region of interest was centred around the hip joint, to be as small as possible to include all the beads in the pelvis superiorly (in the ilium), medially (in the pubis), and the lesser trochanter inferiorly, resulting in an exposure length of around 16.7 cm. Scans were performed with tube potential 100 kVp, tube current 20 mA, slice thickness 0.6 mm and slice increment 0.6 mm, resulting in a voxel size of 0.34 mm × 0.34 mm × 0.6 mm. This CT protocol (Figure 3) resulted in scans consisting of 279 slices with a CT dose index volume (CTDIvol) of 1.05 mGy and a dose length product (DLP) of 17.5 mGycm. Using the conversion factor (k) for the pelvis recommended by the American Association of Physicists in Medicine of 0.015^22^ this gave an estimated effective dose (E) of 0.26 mSv, where E = k*DLP, though actual radiation dose depends on patient characteristics^23^ and individual effective doses will be reported alongside the clinical results. When 10 consecutive patients who had this ceramic HRA implanted were scanned using the low dose CT protocol used in this study as part of an ethically-approved clinical trial (clinicaltrials.gov NCT03326804), their mean effective dose was 0.31 ± 0.02 mSv, which represents a small increase compared to the experimental dose estimate.

### Relative movement measurement

A semi-automated algorithm, written in Matlab (R2018a, The Mathworks, Inc.) was used to compare the relative position of implant and beads between a ‘reference’ scan and the ‘target’ scan, to produce a measurement of relative translation and rotation. The algorithm consisted of the following stages:

#### Segmentation

Beads and implants were segmented from the two CT scans (Mimics v17.0, Materialise, Leuven, Belgium). The mating implant (e.g. the cup implant, when analysing migration of the head implant) and any artefact (defined as scatter radiating from the implant surface) were removed from the model. Operators were trained to recognise artefact. The segmented bead masks were exported as grey-values and coordinates describing each voxel. Beads were 1 mm in diameter. Given the voxel size was 0.34 mm × 0.34 mm × 0.6 mm, a bead would typically be represented as several adjacent voxels, but the segmented mask of voxels would not be spherical. The use of grey-values allowed the centre of the bead to be determined based on the spatial weighting of relative grey-values, rather than simply fitting a 1 mm diameter sphere to the voxels. The implant was exported as a mesh.

#### Bead location

The beads were converted into spherical bead centres, with each bead represented as a single XYZ coordinate. The centre of each bead was calculated as the centre of mass of the weighted grey values in each cluster of masked voxels.

#### Implant matching

Implant meshes from the reference and target scans were manually visually aligned to provide a robust starting point for matching their surfaces (3-matic v9.0, Materialise). An operator-independent automated iterative closest point algorithm (Matlab R2018a, Mathworks) was then applied to finely match the two implants to achieve a best fit.

#### Bead matching

The bead centres from the reference and target scans were introduced and automatically repositioned to align with the new implant positions. An operator-independent iterative closest point algorithm^24^ was used to define the transformation between the two sets of paired bead centres. The condition number, a measure of the appropriateness of the marker distribution,^25^ was reported and monitored.

#### Movement decomposition

The transformation between bead centres from the reference and target scans was decomposed into three translations relative to the centre of the implant, and three rotations about the centre of rotation of the motion stage. In a clinical examination, this would represent the movement (migration) of an implant in relation to fixed beads.

#### Frames of reference

The position of the rigid body of markers in relation to the implant was reported as six parameters, representing six degrees of freedom: three translations along the X (proximal-distal), Y (medial-lateral) and Z (anterior-posterior) axes in mm, and 3 rotations about these axes in Euler angles.

### Precision

The precision of the CTSA method was calculated by performing double measurements – two consecutive CT scans of the phantom with no imposed displacement of the implant – and calculating any difference in implant position. Precision is presented as the 1.96x the standard deviation of these calculated measurements.^26^ A reference scan followed by nine double measurement scans were first performed with the phantom set up to measure head migration, and then repeated with the phantom set up to measure cup migration. After each scan, the phantom was removed from within the CT gantry and placed back in approximately the same position to mimic a clinical double measurement. Two investigators (KL and SGC) independently applied the CTSA method to assess inter-observer reproducibility.

### Accuracy

Accuracy was determined by comparing the CTSA measured movement to defined rig movements.^26^ A reference scan and then a displacement protocol of 17 imposed translations and then 15 rotations was performed (Supplemental Appendix A); first with the phantom set up to simulate head migration and then repeated with it set up to simulate cup migration. The range of migrations used were chosen to represent potential implant migration magnitudes. For translations, the increments increased between 100 µm and 1 mm in the X, Y and Z directions. For rotations, the increments increased between 0.2° and 2° around the X-axis, and between 0.17° and 2° in the Y- and Z-axes. There were no combined translations and rotations. The phantom as a whole was not removed and repositioned in between CT scans. Accuracy was calculated as the 1.96x root mean squared error between the imposed and measured values.

### Statistical analysis

The normality of data was assessed with the Shapiro-Wilk test. Bland-Altman plots were constructed to visually show the agreement between the imposed and measured values and any systematic bias in the CTSA method. The average of the imposed and measured migration is plotted on the X-axis, and the signed error (the difference between the imposed and measured migration) is plotted on the Y-axis. Reproducibility of the method between two observers was measured by the F-statistic of analysis of variance from the expected zero error with a *p*-value < 0.05 considered significant. Statistical analyses were performed using Prism v8.0 (GraphPad, GraphPad Software, La Jolla, CA, USA).

## Results

### Bead placement

The condition numbers of the beads in the pelvis and femur were 16 and 20 respectively, well below 120, which was judged to be acceptable.^25^

### Precision

The precision of the CTSA method was measured separately for head and cup implant migration in 10 consecutive scans with no imposed migration, allowing for nine double-measurement comparisons. The precision (defined as 1.96 × SD) for translation of the head implant ranged from 0.11 to 0.28 mm and for rotation of the head ranged from 0.34° to 0.42° (Table 1). The precision for translation of the cup implant ranged from 0.08 to 0.11 mm and for rotation of the cup ranged from 0.19° to 0.42° (Table 1).

**Table 1.** Precision of CTSA method for measuring zero migration on the phantom pelvis.

Implant	Rig direction	Direction of implant migration	Mean signed error	Precision (1.96 × SD)
Head	X	Superior-inferior (mm)	−0.052	0.278
	Y	Medial-lateral (mm)	0.029	0.267
	Z	Anterior-posterior (mm)	0.012	0.111
	XX	Flexion-extension (°)	0.066	0.412
	YY	Internal-external rotation (°)	0.013	0.423
	ZZ	Varus-valgus (°)	0.058	0.342
Cup	X	Superior-inferior (mm)	0.009	0.105
	Y	Medial-lateral (mm)	0.000	0.076
	Z	Anterior-posterior (mm)	0.012	0.092
	XX	Anterior-posterior tilt (°)	−0.002	0.187
	YY	Internal-external rotation (°)	−0.029	0.384
	ZZ	Adduction-abduction (°)	−0.025	0.420

### Accuracy

**Figure 4.**
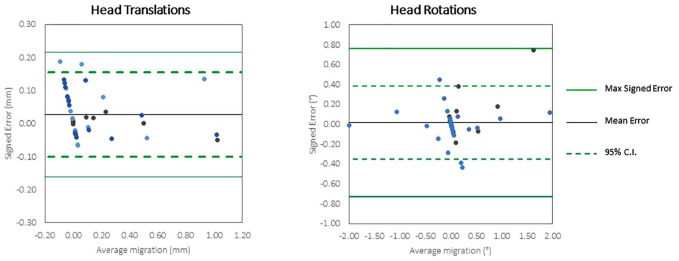
Bland Altman plots of the accuracy of very low dose CTSA to measure translations and rotations of the head implants.

**Figure 5.**
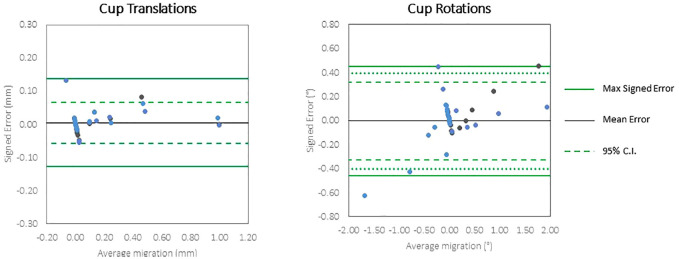
Bland Altman plots of the accuracy of very low dose CTSA to measure translations and rotations of the cup implants.

The accuracy of the CTSA method was measured in 32 scans for head migration (17 translations and 15 rotations) and 31 scans for cup migration (Table 2). The final cup scan with an imposed rotation of 2° in YY was not analysed due to movement of the markers within the phantom. The accuracy (defined as 1.96 × RMS error) for translation of the head implant ranged from 0.043 to 0.181 mm and for rotation of the head ranged from 0.280° to 0.455°. The accuracy for translation of the cup implant ranged from 0.041 to 0.081 mm and for rotation of the cup ranged from 0.168° to 0.429°. Bland-Altman plots (Figures 4 and 5) show the spread of error, centred around zero for both cup and head, in translations and rotations.

**Table 2.** Accuracy of CTSA method for imposed migration on the phantom pelvis.

Implant	Rig direction	Direction of implant migration	Mean signed error	SD	Accuracy (1.96 × RMS)
Head	X	Superior-inferior (mm)	0.040	0.068	0.150
	Y	Medial-lateral (mm)	0.044	0.084	0.181
	Z	Anterior-posterior (mm)	−0.002	0.022	0.043
	XX	Flexion-extension (°)	−0.081	0.169	0.358
	YY	Internal-external rotation (°)	0.050	0.138	0.280
	ZZ	Varus-valgus (°)	0.077	0.226	0.455
Cup	X	Superior-inferior (mm)	0.009	0.020	0.041
	Y	Medial-lateral (mm)	0.003	0.043	0.081
	Z	Anterior-posterior (mm)	0.000	0.031	0.059
	XX	Anterior-posterior tilt (°)	−0.073	0.223	0.429
	YY	Internal-external rotation (°)	0.019	0.090	0.168
	ZZ	Adduction-abduction (°)	0.041	0.150	0.285

### Reproducibility

The reproducibility of the CTSA method was evaluated by comparing the error in measurement between two observers (Table 3). The *F*-test to compare variances demonstrated no significant differences between the observers for the cup and head, in translations and rotations.

**Table 3.** Reproducibility of CTSA method, by comparing variances in error between two observers.

Implant	Movements	*F*-statistic	*p*-Value
Head	Translations	1.031	0.886
	Rotations	1.058	0.938
Cup	Translations	1.089	0.551
	Rotations	1.628	0.221

## Discussion

A CT-based spatial analysis method to measure migration of an unmodified all-ceramic HRA was developed and validated. The aim of this method is to be used as an assessment tool in the release of new implants and to be implemented as part of a premarket clinical safety investigation. The benefit of this method over model-based RSA is that there is no requirement for calibrated radiographs, which must be taken with specialist equipment which is not widely available.

Our method includes operator dependent steps, which may influence the results, in particular the segmentation of any beads which are distorted by artefact. In practice this was observed to occur in a minimal number of beads. The intra-observer analysis showed no significant difference between operators. This is likely owing to minimal artefact, low condition number and the high number of beads, which reduce the sensitivity of the bead registration to any individual bead. Removal of artefact during implant segmentation was also operator dependent. In all cases, the majority of the voxels corresponding to the implant remained after artefact removal, and the voxels labelled as artefacts were validated by the observer.

The modification of implants with markers requires authorisation and assistance from manufacturers, may alter implant performance, is costly and in some implants, such as the all-ceramic monobloc HRA used in this study, it is not possible to impregnate beads into the device. Ideally, markers would not be required within the bone either, but CT imaging of monoblock orthopaedic implants results in metal/ceramic artefacts, streak artefacts and scatter,^27^ which gets worse with a lower dose; while higher radiation exposure is associated with cancer.^28^ The hybrid protocol presented here (beads in surrounding bone, with an unmodified implant) is a compromise, respecting the ‘as low as reasonably achievable’ principle of ionising radiation while achieving accuracy and precision comparable to model-based RSA.

Migration measured at 2 years postoperatively is accepted as prognostic of long-term implant survival.^8,26^ There is a dichotomy of migration patterns between implants that are well fixed and those that are at risk of aseptic loosening and subsequent failure.^2,5,29,30^ Therefore, thresholds for migration can be used for assessment of new prostheses.^31^ Meta-analysis by matching 2-year RSA migration of prostheses with 10-year survival data showed that for the acetabular cup implant, 1 mm of proximal migration after 2 years is an unacceptable limit denoting an implant likely to loosen.^2^ Given the accuracy of our CTSA method, it could be used in longitudinal assessment to identify cups with unacceptable migration. No acceptability threshold has so far been established for HRA femoral head implants.

Our findings should be viewed in the context of limitations. Firstly, this method was validated in an experimental setting using a specific implant and not accounting for soft tissue; but precision measured in a phantom study has been shown to be similar to that measured in a clinical setting, when the same protocol settings are used.^16^ Secondly, the model-based method described in this study matched the contoured rims of the implants between two scans and used the beads embedded in the pelvic model to create a frame of reference. Due to artefact from ceramic attenuation in the plane of the x-ray beam, not all the contour could be used for matching. Slices with both head and cup have more ceramic and thus more artefact in them than slices with head only or cup only. Both the precision and accuracy of translations in X and Y axes are higher (indicated by a lower error) in the cup than the head implant. Given the nature of a hip arthroplasty implant (with the head sitting inside the cup), there is more unattenuated slice data containing the cup only, and thus more surface area to enable shape matching between scans. This explains the lower error and also highlights the importance of validating the method with both implants in situ.

Reported precisions (when defined as 1.96 × SD) for traditional and model-based RSA in comparable acetabular cup configurations range from 0.01 to 0.29 mm for translations and from 0.05° to 0.59° for rotations.^9,10,12,13^ Reported precisions for CT-based migration analysis techniques in acetabular cups range from 0.03 to 0.16 mm for translations and from 0.06° to 0.37° for rotations.^9,10,12^ The method presented in this paper shows comparable acetabular cup precision to these methods of 0.08–0.11 mm for translations and 0.19°–0.42° for rotations.

One study reported the precision (when defined as 1.96 × SD) of traditional and model-based RSA for a resurfacing head ranging from 0.04 to 0.82 mm for translations and from 0.10° to 2.20° for rotations.^15^ The method presented in this paper shows comparable resurfacing head precision to these methods of 0.11–0.28 mm for translations and 0.34°–0.42° for rotations.

The reported accuracy for RSA in other joints varies between 0.10 and 0.70 mm for translations and 0.03° and 0.5° for rotations.^6–8^ Brodén et al. undertook a phantom study, similar to the one reported here, and found accuracy (defined as 2.57 × RMS) ranging from 0.08 to 0.32 for translations and from 0.21° to 0.82° for rotations for cemented and uncemented acetabular cups.^12^ The method presented in the current study shows comparable acetabular cup accuracy (defined as 1.96 × RMS) of 0.04–0.08 mm for translations and 0.17°–0.43° for rotations.

This study used a low dose CTSA method to measure both imposed translations and rotations, in femoral and acetabular hip resurfacing implants without modification with both implants in situ. This method, along with other CT-based motion analysis methods, has significant advantages over the current gold-standard, RSA. CTSA uses an implant-based coordinate system and an unmodified hospital CT scanner. It enables fast acquisition without the need for precise patient positioning or additional calibration cages or radiographer expertise. It should be recognised, however, that some expertise and 10–15 min is required to perform the manual steps described here for each image pair, including image registration and analysis.

## Conclusions

This in vitro study demonstrates that CTSA can reliably measure simulated implant migration in a ceramic monobloc HRA implant using a low dose CT protocol and does so with an accuracy and precision that is similar to both RSA and other CTMA techniques. CT is ubiquitous, so this may be a more feasible method to measure migration of an unmodified prosthesis after arthroplasty in multicentre preclinical studies than RSA. Having established the feasibility, accuracy and precision of this CTSA method in an experimental setting, our current clinical work is investigating its validity to measure migration in patients as part of a premarket clinical safety investigation.

## Supplemental Material

sj-docx-1-pih-10.1177_09544119231153905 – Supplemental material for Low dose CT-based spatial analysis (CTSA) to measure implant migration after ceramic hip resurfacing arthroplasty (HRA): A phantom study Click here for additional data file. Supplemental material, sj-docx-1-pih-10.1177_09544119231153905 for Low dose CT-based spatial analysis (CTSA) to measure implant migration after ceramic hip resurfacing arthroplasty (HRA): A phantom study by Susannah G Clarke, Kartik Logishetty, Camilla Halewood and Justin P Cobb in Proceedings of the Institution of Mechanical Engineers, Part H: Journal of Engineering in Medicine

## References

[1] NJR. National joint registry annual report. Report no. 19, 2022. NJR. 38422195

[2] Pijls BG Nieuwenhuijse MJ Fiocco M , et al. Early proximal migration of cups is associated with late revision in THA: a systematic review and meta-analysis of 26 RSA studies and 49 survivalstudies. Acta Orthop 2012; 83: 583–591. 23126575 10.3109/17453674.2012.745353 PMC3555453

[3] Derbyshire B Prescott RJ Porter ML. Notes on the use and interpretation of radiostereometric analysis. Acta Orthop 2009; 80: 124–130. 19234894 10.1080/17453670902807474 PMC2823227

[4] Abrahams JM Callary SA Jang SW , et al. Accuracy of EBRA-cup measurements after reconstruction of severe acetabular defects at revision THR. J Orthop Res 2020; 38: 1497–1505. 32039492 10.1002/jor.24623

[5] van der Voort P Pijls BG Nieuwenhuijse MJ , et al. Early subsidence of shape-closed hip arthroplasty stems is associated with late revision. A systematic review and meta-analysis of 24 RSA studies and 56 survival studies. Acta Orthop 2015; 86: 575–585. 25909455 10.3109/17453674.2015.1043832 PMC4564780

[6] Selvik G. Roentgen stereophotogrammetry. A method for the study of the kinematics of the skeletal system. Acta Orthop Scand Suppl 1989; 232: 1–51. 2686344

[7] Ryd L. Roentgen stereophotogrammetric analysis of prosthetic fixation in the hip and knee joint. Clin Orthop Relat Res 1992; 276: 56–65. 1537175

[8] Kärrholm J Herberts P Hultmark P , et al. Radiostereometry of hip prostheses. Review of methodology and clinical results. Clin Orthop Relat Res 1997; 344: 94–110. 9372762

[9] Angelomenos V Mohaddes M Itayem R , et al. Precision of low-dose CT-based micromotion analysis technique for the assessment of early acetabular cup migration compared with gold standard RSA: a prospective study of 30 patients up to 1 year. Acta Orthop 2022; 93: 459–465. 35478261 10.2340/17453674.2022.2528 PMC9047498

[10] Brodén C Sandberg O Olivecrona H , et al. Precision of CT-based micromotion analysis is comparable to radiostereometry for early migration measurements in cemented acetabular cups. Acta Orthop 2021; 92: 419–423. 33821746 10.1080/17453674.2021.1906082 PMC8381926

[11] Otten V Maguire GQ Jr Noz ME , et al. Are CT scans a satisfactory substitute for the follow-up of RSA migration studies of uncemented cups? A comparison of RSA double examinations and CT datasets of 46 total hip arthroplasties. Biomed Res Int 2017; 2017: 3681458. 28243598 10.1155/2017/3681458 PMC5294349

[12] Brodén C Olivecrona H Maguire GQ Jr , et al. Accuracy and precision of three-dimensional low dose CT compared to standard RSA in acetabular cups: an experimental study. Biomed Res Int 2016; 2016: 5909741. 27478832 10.1155/2016/5909741 PMC4958415

[13] Shareghi B Johanson PE Kärrholm J. Clinical evaluation of model-based radiostereometric analysis to measure femoral head penetration and cup migration in four different cup designs. J Orthop Res 2017; 35: 760–767. 26825861 10.1002/jor.23177

[14] Seehaus F Emmerich J Kaptein BL , et al. Dependence of model-based RSA accuracy on higher and lower implant surface model quality. Biomed Eng Online 2013; 12: 32. 23587251 10.1186/1475-925X-12-32 PMC3637620

[15] Lorenzen ND Stilling M Jakobsen SS , et al. Marker-based or model-based RSA for evaluation of hip resurfacing arthroplasty? A clinical validation and 5-year follow-up. Arch Orthop Trauma Surg 2013; 133: 1613–1621. 24100765 10.1007/s00402-013-1850-2

[16] Eriksson T Maguire GQ Noz ME , et al. Are low-dose CT scans a satisfactory substitute for stereoradiographs for migration studies? A preclinical test of low-dose CT scanning protocols and their application in a pilot patient. Acta Radiol 2019; 60: 1643–1652. 31042065 10.1177/0284185119844166

[17] Olivecrona H Maguire GQ Noz ME , et al. A CT method for following patients with both prosthetic replacement and implanted tantalum beads: preliminary analysis with a pelvic model and in seven patients. J Orthop Surg Res 2016; 11: 27. 26911571 10.1186/s13018-016-0360-7 PMC4766687

[18] Scheerlinck T Polfliet M Deklerck R , et al. Development and validation of an automated and marker-free CT-based spatial analysis method (CTSA) for assessment of femoral hip implant migration in vitro accuracy and precision comparable to that of radiostereometric analysis (RSA). Acta Orthop 2016; 87: 139–145. 26634843 10.3109/17453674.2015.1123569 PMC4812075

[19] Lindgren L Jørgensen PB Mørup RMS , et al. Similar patient positioning: A key factor in follow-up studies when using model-based radiostereometric analysis of the hip. Radiography 2020; 26: E45–E51. 32052775 10.1016/j.radi.2019.10.009

[20] Nazari-Farsani S Finnilä S Moritz N , et al. Is model-based radiostereometric analysis suitable for clinical trials of a cementless tapered wedge femoral stem? Clin Orthop Relat Res 2016; 474(10): 2246–2253. 27334320 10.1007/s11999-016-4930-0 PMC5014820

[21] Baad-Hansen T Kold S Kaptein BL , et al. High-precision measurements of cementless acetabular components using model-based RSA: an experimental study. Acta Orthop 2007; 78: 463–469. 17965999 10.1080/17453670710014095

[22] AAPM. The measurement, reporting, and management of radiation dose in CT. Report no. 96, 2008. American Association of Physicists in Medicine.

[23] Shrimpton PC Hillier MC Lewis MA , et al. Doses from computed tomography (CT) examinations in the UK - NRPB-W67. Report no. NRPB-W67, 2003. Chilton: National Radiological Protection Board.

[24] Chen Y Medioni G. Object modelling by registration of multiple range images. Image Vis Comput 1992; 10: 145–155.

[25] Valstar ER Gill R Ryd L , et al. Guidelines for standardization of radiostereometry (RSA) of implants. Acta Orthop 2005; 76: 563–572. 16195075 10.1080/17453670510041574

[26] ISO 16087:2013. Implants for surgery – roentgen stereophotogrammetric analysis for the assessment of migration of orthopaedic implants.

[27] Boas FE Fleischmann D. CT artifacts: causes and reduction techniques. Imaging Med 2012; 4: 229–240.

[28] Shuryak I Sachs RK Brenner DJ. Cancer risks after radiation exposure in middle age. J Natl Cancer Inst 2010; 102: 1628–1636. 20975037 10.1093/jnci/djq346 PMC2970575

[29] Pijls BG Plevier JWM Nelissen RGHH . RSA migration of total knee replacements. Acta Orthop 2018; 89: 320–328. 29508661 10.1080/17453674.2018.1443635 PMC6055769

[30] Pijls BG Valstar ER Nouta KA , et al. Early migration of tibial components is associated with late revision: a systematic review and meta-analysis of 21,000 knee arthroplasties. Acta Orthop 2012; 83: 614–624. 23140091 10.3109/17453674.2012.747052 PMC3555454

[31] Malchau H Bragdon CR Muratoglu OK. The stepwise introduction of innovation into orthopedic surgery: the next level of dilemmas. J Arthroplasty 2011; 26: 825–831. 20888183 10.1016/j.arth.2010.08.007

