# Supplementary material for: Low dose CT-based spatial analysis (CTSA) to measure implant migration after ceramic hip resurfacing arthroplasty (HRA): A phantom study
Source: Proc Inst Mech Eng H. 2023 Feb 11;237(3):359–67. doi: 10.1177/09544119231153905 (PMC10052406; doi:10.1177/09544119231153905)
Supplement: sj-docx-1-pih-10.1177_09544119231153905 – Supplemental material for Low dose CT-based spatial analysis (CTSA) to measure implant migration after ceramic hip resurfacing arthroplasty (HRA): A phantom study [file sj-docx-1-pih-10.1177_09544119231153905.docx]

**Appendix A: Imposed translations and rotations**

**Table 1: Imposed translations in the X, Y and Z axes, performed with a fixed femur and a fixed pelvis, to simulate cup and head migration**

| **Scan** | **Translations (mm)** | | |
| --- | --- | --- | --- |
|  | **X** | **Y** | **Z** |
| 1 | 0.1 | 0 | 0 |
| 2 | 0.15 | 0 | 0 |
| 3 | 0.25 | 0 | 0 |
| 4 | 0.5 | 0 | 0 |
| 5 | 1 | 0 | 0 |
| 6 | 0 | 0.1 | 0 |
| 7 | 0 | 0.15 | 0 |
| 8 | 0 | 0.25 | 0 |
| 9 | 0 | 0.5 | 0 |
| 10 | 0 | 1 | 0 |
| 11 | 0 | 0 | 0.1 |
| 12 | 0 | 0 | 0.15 |
| 13 | 0 | 0 | 0.25 |
| 14 | 0 | 0 | 0.5 |
| 15 | 0 | 0 | 1 |
| 16 | 0.1 | 0 | 0 |
| 17 | 0.15 | 0 | 0 |

**Table 2: Imposed rotations in the X, Y and Z axes, performed with a fixed femur and a fixed pelvis, to simulate cup and head migration**

| **Scan** | **Rotations (°)** | | |
| --- | --- | --- | --- |
|  | **X** | **Y** | **Z** |
| 1 | -0.2 | 0 | 0 |
| 2 | -0.32 | 0 | 0 |
| 3 | -0.48 | 0 | 0 |
| 4 | -1 | 0 | 0 |
| 5 | -2 | 0 | 0 |
| 6 | 0 | 0.17 | 0 |
| 7 | 0 | 0.33 | 0 |
| 8 | 0 | 0.5 | 0 |
| 9 | 0 | 1 | 0 |
| 10 | 0 | 2 | 0 |
| 11 | 0 | 0 | 0.17 |
| 12 | 0 | 0 | 0.33 |
| 13 | 0 | 0 | 0.5 |
| 14 | 0 | 0 | 1 |
| 15 | 0 | 0 | 2 |
